# *In silico* analysis to develop PCR assays for identification of bacterial pathogens in animals: what can we improve?

**DOI:** 10.3389/fvets.2023.1235837

**Published:** 2023-08-14

**Authors:** Alexandra Bogomazova, Ekaterina Krylova, Irina Soltynskaya, Olga Prasolova, Olga Ivanova

**Affiliations:** ^1^Department of Molecular Biology, Russian State Center for Quality and Standardization of Veterinary Drugs and Feed (VGNKI), Moscow, Russia; ^2^Laboratory of Cell Biology, Lopukhin Federal Research and Clinical Center of Physical-Chemical Medicine of FMBA of Russia (Lopukhin FRCC PCM), Moscow, Russia

**Keywords:** PCR assay, bacterial pathogens, nucleotide collection database, *in silico* analysis, assay development

## Introduction

Polymerase chain reaction (PCR) as a diagnostic method has gained enormous notability during the coronavirus pandemic (1). Indeed, PCR is an indispensable tool for rapidly assessing the spread of a disease spreading during viral infection outbreaks in the human population, animal herds and poultry. More generally, PCR is one of the primary methods for diagnostics of viral infections in clinical and veterinary medicine.

Regarding bacterial pathogens, PCR-based diagnostics play a more modest role since laboratories rely mostly on culture-based methods (2). Nevertheless, the World Organization for Animal Health (WOAH, founded as OIE) recommends the use of PCR assay as a supplement to traditional diagnostic methods for *Mycoplasma spp*., *Mycobacterium tuberculosis, Pasteurella multocida* and *Chlamydia spp*. For example, recommendations to employ genetic tests are present in the WOAH guidelines for the diagnostics of mycoplasmosis (3), tuberculosis (4), fowl cholera (5), and chlamydiosis (6). However, no WOAH diagnostics guidelines have yet been developed for contagious diseases, such as infectious coryza, bordetellosis, ornitobacteriosis, serositis, etc.

Some bacterial pathogens that cause the above diseases are difficult to culture *in vitro*. For instance, *Ornithobacterium rhinotracheale*, which causes bird ornitobacteriosis, and *Avibacterium paragallinarum*, which causes infectious coryza are very demanding to culture conditions (7, 8). In addition, frequent mixed infections can seriously hamper the identification of pathogens (9). For such diseases, the PCR assay can become a primary diagnostic method. Furthermore, the PCR-based identification of pathogens circulating in an animal population can also be helpful in the choosing a vaccination strategy (10). Indeed, papers on the development and validation of novel PCR-based diagnostic assays for bacterial pathogens are regularly published in veterinary journals.

When designing PCR assays, developers follow a standard workflow consisting of three main stages: *in silico* stage, the evaluation of analytical performance of PCR, and evaluation of its diagnostic performance (11, 12). An *in silico* bioinformatics analysis involves the use of computational tools and databases to check the quality of designed PCR primers. By performing the *in silico* bioinformatics analysis, potential problems such as primer-dimer formation, hairpin formation, primer mispriming, and false negative or false positive results can be avoided. Here, we briefly review the *in silico* stage of the recently published PCR assays, focusing on how the authors account for genetic polymorphism that can cause false negative results in a PCR assay. For this mini-review, we compared nearly three dozen real-time PCR diagnostic assays developed in the last 5 years to identify bacteria causing upper respiratory tract diseases in animals (Table 1). We considered only those PCR assays that assessed the diagnostic performance of PCR tests. In the end, we formulate some suggestions for improving the presentation of *in silico* analysis in publications.

**Table 1:** List of PCR assays reviewed in this article.

**Reference**	**Pathogen**	**Gene**	**Sequence collections used in polymorphism analysis**	Polymorphic primer sites^**^	**Number of sequences in NCBI collections (as of May, 2023)**		
					**nt/nr**	**Refseq genomes**	**wgs**
Arai et al. (13)	*Streptococcus parasuis*	*recN*	nt/nr	+	**26** ^*^	10	10
Hashish et al. (7)	*Ornithobacterium rhinotracheale*	*16S rRNA*	–	+	**284**	20	9
Hashish et al. (14)	*Bordetella avium*	*BAV1945*	nt/nr	–	6	**26**	20
Hashish et a. (14)	*Bordetella avium*	*fhaC*	nt/nr	–	7	**26**	20
Goecke et al. (15)	*Trueperella pyogenes*	*plo*	–	+	**51**	**27**	10
Sunaga et al. (16)	*Mesomycoplasma hyopneumoniae*	*p102*	–	–	13	**28**	14
Kuchipudi et al. (8)	*Avibacterium paragallinarum*	*recN*	nt/nr, wgs	–	17	**50**	34
Loy et al. (17)	*Histophilus somni*	*ompA*	refseq genomes	+	31	**59**	35
Goecke et al. (15)	*Histophilus somni*	*16S rRNA*	–	+	**105**	60	35
Sunaga et al. (16)	*Mycoplasma hyosynoviae*	*rpoB*	–	+	7	**73**	69
Srijuntongsir et al. (18)	*Actinobacillus pleuropneumoniae*	*omlA (apxIVA)*	nt/nr, wgs	–	57	**78**	49
Srijuntongsir et al. (18)	*Actinobacillus pleuropneumoniae*	*eamA*	nt/nr, wgs	–	36	**78**	49
Srijuntongsir et al. (18)	*Actinobacillus pleuropneumoniae*	*nusG*	nt/nr, wgs	–	36	**78**	49
Srijuntongsir et al. (18)	*Actinobacillus pleuropneumoniae*	*sppA*	nt/nr, wgs	–	36	**78**	49
Srijuntongsir et al. (18)	*Actinobacillus pleuropneumoniae*	*xerD*	nt/nr, wgs	–	36	**78**	49
Srijuntongsir et al. (18)	*Actinobacillus pleuropneumoniae*	*ybbN*	nt/nr, wgs	–	36	**78**	49
Srijuntongsir et al. (18)	*Actinobacillus pleuropneumoniae*	*ycfL*	nt/nr, wgs	+	36	**78**	49
Srijuntongsir et al. (18)	*Actinobacillus pleuropneumoniae*	*ychJ*	nt/nr, wgs	–	36	**78**	49
Sunaga et al. (16)	*Actinobacillus pleuropneumoniae*	*omlA (apxIVA)*	–	+	57	**78**	49
Sunaga et al. (16)	*Mycoplasma hyorhinis*	*p37*	–	–	31	**85**	85
Sunaga et al. (16)	*Bordetella bronchiseptica*	*flaA*	–	–	39	**96**	79
Goecke et al. (15)	*Mycoplasma bovis*	*oppD*	–	+	196	168	**428**
Goecke et al. (15)	*Mannheimia haemolytica*	*sodA*	–	+	108	212	**1,613**
Loy et al. (17)	*Mannheimia haemolytica*	*lktD*	refseq genomes, (19)	+	121	212	**1,613**
Sunaga et al. (16)	*Haemophilus parasuis*	*inf B*	–	–	77	219	**431**
Goecke et al. (15)	*Pasteurella multocida*	*kmt1*	–	+	152	407	**882**
Loy et al. (17)	*Pasteurella multocida*	*Pm1231*	refseq genomes	+	152	407	**882**
Sunaga et al. (16)	*Pasteurella multocida*	*kmt1*	–	+	215	407	**882**
Arai et al. (13)	*Streptococcus suis*	*recN*	nt/nr	+	172	1,989	**2,966**
Sunaga et al. (16)	*Streptococcus suis*	*16S RNA*	–	+	413	1,989	**2,966**

^*^A priority database for consideration of polymorphism in the bacterial gene is indicated in bold.

^**^The analysis for polymorphic primer sites was conducted with the NCBI collections accessed in May, 2023.

## Common practice for *in silico* analysis of genetic polymorphism to develop PCR assays

Intra-species genetic variation can compromise the diagnostic sensitivity of PCR, increasing the likelihood of false negative results. For instance, genetic polymorphism can seriously impair primer annealing, especially if a variable nucleotide is at the 3′ end of the primer. Moreover, the impact of genetic polymorphism on PCR efficiency can be even more significant if the polymorphic position is at the annealing site of the PCR probe. Guidelines for creating diagnostic PCR tests recommend taking genetic polymorphism into account during the assay development (20), but this recommendation is not specific enough. For example, there are no quantitative endpoints for considering genetic polymorphisms. There are also no guidelines for presenting the results of the *in silico* analysis of genetic polymorphisms.

Not surprisingly, the authors describe the *in silico* analysis with varying levels of detail and depth (Table 1). For example, all authors used the BLAST tool (21) to check how the sequence variation within a bacterial species could affect the PCR sensitivity. However, in many cases they did not specify which NCBI database was searched: the nucleotide collection (nt/nr), the RefSeq genome database (refseq genomes), or whole-genome shotgun database (wgs) (22, 23). Thus, the BLAST search coverage was not clear in many cases.

Moreover, the authors were choosing different NCBI collections for analysis of genetic polymorphisms (Table 1). Most likely, the choice depended on personal preferences. However, the choice of the database may have depended on the target gene and the number of genomes for a bacterial species in the NCBI collections. In our sample, nearly 40 percent of the PCR target gene sequences were submitted to the NCBI sequence collections as a result of whole genome sequencing. However, other target genes, including phylogenetic genetic markers (*16S rRNA, recN*), virulence genes (*kmt1, oppD, omlA*), and multilocus sequence typing gene (*infA*) were submitted to the nt/nr database as result of Sanger sequencing. Therefore, for these genes, intra-species polymorphism may be more widely represented in the nt/nr database compared to the wgs database. For instance, the *Streptococcus parasuis recN* gene used as a target gene by Arai et al. (13) is a phylogenetic marker with 26 sequences in the nt/nr database and only 10 sequences in the wgs database. On the other hand, the wgs database is the most representative for bacterial species, the whole genome of which has been sequenced more than 150 times. The refseq genomes database is optimal for bacterial species with intermediate number of genomes sequenced (Table 1). It should be emphasized that NCBI Genbank collections may not accurately reflect true genetic polymorphism due to uneven representation of different regions, annotation inaccuracies, or technical sequencing errors. So, *in silico* analysis should always be accompanied by verification of PCR sensitivity and specificity on real samples.

Loy et al. (17) provided an excellent example of how to take genetic polymorphism into account. In their study, degenerate nucleotides were placed at polymorphic positions in the primer and multiple alignments were used to illustrate variable positions. However, most authors did not explicitly state whether polymorphic nucleotides were present at the primer annealing sites. As a result, we examined the primer annealing sites for polymorphism in all PCR tests listed in Table 1. In May 2023, we accessed the NCBI sequence collections, the number of sequences of which has increased significantly since the publication of the analyzed primers. We found polymorphic positions in 50% of the cases, which was common among bacterial species with the highest numbers of genomes in wgs database. However, *the sodA* (15) and *lktD* (17) genes were found to be very conservative with single-variant sequences among 1,613 *Mannheimia haemolytica* genomes in the wgs database. The frequency of *sodA* and *lktD* sequences with a polymorphic nucleotide in the primer was only 0.06%. In other words, the primers and probes perfectly matched the target sequence in 99.94% of the *Mannheimia haemolytica* genomes. It should also be noted that a BLAST search in the wgs database allows detection if a target PCR sequence is not ubiquitous for the genome sequenced of a given bacterial species. For instance, the wgs database comprises 1,613 genomes of *Mannheimia haemolytica* and the target sequence for PCR in the *sodA* gene (15) is present in 1,611 cases.

## Conclusion

The NCBI sequence collections now contain thousands of genomes for a variety of pathogenic bacteria, as shown in Table 1. The abundance of data allows for comprehensive testing of primers for many sequences. On the other hand, the growth of the NCBI sequence collections will inevitably make it increasingly difficult for researchers to find perfectly conservative sites for primer design. This highlights the need for establishing pipelines that take into account genetic polymorphism when developing PCR assays for diagnostics of bacterial pathogens.

We believe that developers should clearly state the sequence database used with the BLAST tool and provide the number of bacterial gene sequences available for analysis. The presence or absence of polymorphism at the annealing sites of primers and samples should be clearly indicated. If polymorphic sites are present, it is appropriate to demonstrate the polymorphism using a multiple sequence alignment, as shown by Loy et al. (17) in the supplemental material to their publication. Moreover, a quantitative assessment may be included, such as “…the primers and probe perfectly match 99% of the genomic sequences of the target species deposited in the NCBI reference genome database…” When using previously published primers, it is important to recheck them for the presence of polymorphic sites at the annealing sites of primers and probe in current sequence databases. In addition, the authors should disclose if the PCR target sequence is absent in some bacterial genomic sequences deposited to the NCBI collections.

## Author contributions

AB conceptualized the study and wrote the manuscript. EK and IS conducted literature analysis and searched the NCBI database. OP analyzed the manuals of diagnostic tests for terrestrial animals. OI secured funding for the work. All authors contributed to the article and approved the final version for submission.
